# Imaging of Skull Base and Orbital Invasion in Sinonasal Cancer: Correlation with Histopathology

**DOI:** 10.3390/cancers13194963

**Published:** 2021-10-01

**Authors:** Maxime Salfrant, Gabriel C. T. E. Garcia, Jean-Pierre Guichard, François Bidault, Daniel Reizine, Anne Aupérin, Damien Bresson, Benjamin Verillaud, Philippe Herman, Antoine Moya-Plana

**Affiliations:** 1 Otorhinolaryngology and Skull Base Center, AP-HP, Lariboisière Hospital, 75010 Paris, France; maxime.salfrant@aphp.fr (M.S.); benjamin.verillaud@aphp.fr (B.V.); philippe.herman@aphp.fr (P.H.); 2 Radiology Department, Gustave Roussy cancer Campus, 94805 Villejuif, France; GABRIEL.GARCIA@gustaveroussy.fr (G.C.T.E.G.); francois.bidault@gustaveroussy.fr (F.B.); 3 Radiology Department, AP-HP, Lariboisière Hospital, 75010 Paris, France; jean-pierre.guichard@aphp.fr (J.-P.G.); daniel.reizine@aphp.fr (D.R.); 4 Biostatistical and Epidemiological Division, Gustave Roussy cancer Campus, 94805 Villejuif, France; anne.auperin@gustaveroussy.fr; 5 Neurosurgery Department, AP-HP, Henri Mondor Hospital, 94000 Créteil, France; damien.bresson@aphp.fr; 6 Head and Neck Oncology Department, Gustave Roussy cancer Campus, 94805 Villejuif, France

**Keywords:** orbit, paranasal sinus neoplasm, radiology, skull base, surgery

## Abstract

**Simple Summary:**

Pretreatment assessment of local extension in sinonasal cancer is essential for prognostic evaluation and surgical planning. It essentially relies on CT and MRI imaging whose performance is not accurately described in the scientific literature. The aim of this study was to assess the diagnostic performance of CT and MRI for the diagnosis of skull base and orbital invasion in sinonasal cancer by comparing imaging findings to histopathological data. A total of 176 patients were included. Objective data about the diagnostic value of pretreatment imaging in patients with sinonasal cancer were obtained: they suggest that pretreatment assessment of orbital invasion is difficult, even with the combination of CT and MRI.

**Abstract:**

Background: Pretreatment assessment of local extension in sinonasal cancer is essential for prognostic evaluation and surgical planning. The aim of this study was to assess the diagnostic performance of two common imaging techniques (CT and MRI) for the diagnosis of skull base and orbital invasion by comparing imaging findings to histopathological data. Methods: This was a retrospective two-center study including patients with sinonasal cancer involving the skull base and/or the orbit operated on between 2000 and 2019. Patients were included only if pre-operative CT and/or MRI, operative and histopathologic reports were available. A double prospective blinded imaging review was conducted according to predefined radiological parameters. Radiologic tumor extension was compared to histopathological reports, which were considered the gold standard. The predictive positive value (PPV) for the diagnosis of skull base/orbital invasion was calculated for each parameter. Results: A total of 176 patients were included. Ethmoidal intestinal-type adenocarcinoma was the most common type of cancer (41%). The PPV for major modification of the bony skull base was 78% on the CT scan, and 89% on MRI. MRI signs of dural invasion with the highest PPVs were: contact angle over 45° between tumor and dura (86%), irregular deformation of dura adjacent to tumor (87%) and nodular dural enhancement over 2 mm in thickness (87%). Signs of orbital invasion had low PPVs (<50%). Conclusions: This retrospective study provides objective data about the diagnostic value of pretreatment imaging in patients with sinonasal cancer.

## 1. Introduction

Sinonasal cancers are rare, accounting for 3% of all head and neck malignancies. Histology and prognosis are highly variable for these tumors. Their treatment is generally based on surgery followed by radiotherapy [1]. Pretreatment imaging by a CT scan and MRI is essential to assess the local extension of the tumor [2] and to determine its resectability, massive cerebral invasion and/or invasion of vascular structures such as the internal carotid artery or cavernous sinus, being a contraindication for surgical treatment. It also guides the choice of the surgical approach and the planning of the surgical steps. Currently, endoscopic endonasal resection is preferred to the historical craniofacial resection, considering its reliability with comparable oncologic results and a lower morbidity [3]. The radiological assessment of the tumor origin is a crucial piece of information before endoscopic endonasal surgery, especially for the planification of skull base resection and reconstruction [3]. Contraindications of this technique are often detected by imaging: orbital involvement requiring exenteration, massive dural invasion over orbital roof, invasion of maxillary sinus walls (except for the medial one) [4,5]. In addition, imaging provides key information about the prognosis of the tumor, with orbital and dural invasion representing well-known negative prognostic factors [6,7]. Thus, pretreatment imaging has a major impact on patient care.

However, there are some limitations as macroscopic intraoperative and histopathological findings frequently differ from the extension reported in pre-operative imaging [8,9]. Frozen section analysis is hence a helpful tool to perform oncological resection with clear margins.

Several authors have studied the performance of imaging in sinonasal tumors. CT scans show the best performance for analysis of thin bony structures such as the skull base and orbital walls [10,11,12,13,14]. Lund et al. [15] reported a 78% accuracy between CT scan, operative and histopathologic findings. Other studies correlated radiological assessment of tumor extension with histopathologic findings: they predominantly used small retrospective cohorts. Most of them evaluated skull base and dural invasion [9,15,16,17,18,19,20,21], whereas a few of them evaluated orbital invasion [21,22,23,24]. Double reviewing was not performed in all studies. Therefore, we decided to conduct a retrospective study with independent and blinded prospective double radiological reviewing in two referral centers over a long period of time.

The aim of this study was to assess the diagnostic performance of two common imaging techniques (CT and MRI) for the diagnosis of skull base and orbital invasion in sinonasal cancer by comparing imaging findings to histopathological data.

## 2. Materials and Methods

A retrospective study was conducted in two referral centers for skull base cancers. This study included patients operated on for sinonasal cancer involving the skull base and/or the orbit between January 2000 and July 2019.

A retrospective chart review was performed to collect patients’ information: sex, age at the time of surgery, pre-operative TNM (cTNM) classification of the tumor according to the 8th AJCC edition [25], histologic subtypes, side and primitive anatomical location of the tumor, primary tumor or recurrence, surgical approach (craniofacial, endoscopic trans-nasal or cranio-endoscopic resection), pre- and post-operative treatments (chemotherapy, radiotherapy), delay between CT scan/MRI and surgery.

On histopathological reports, the following information was recorded: post-operative TNM (pTNM) classification according to the 8th AJCC edition, surgical margins, microscopic tumor extension to the bony skull base (ethmoidal roof, cribriform plate, planum), bony orbital walls (lamina papyracea, orbital roof and floor), orbital content (periorbita or fat), dura, olfactory bulb and cerebral parenchyma. We assessed tumor invasion both on permanent surgical specimens and on frozen sections for nasal mucosa, periorbita and dura. When “en-bloc” surgery was feasible and performed, margins were evaluated on operative bed and surgical specimens. If piecemeal resection was realized, which is often the case in skull base surgery, the analysis of additional peripheral and deep margins was mandatory to evaluate the quality of tumor resection [4,26,27]. Pre- and post-operative TNM stages were compared focusing on the primary tumor site and the histologic subtype.

**Figure 1:**
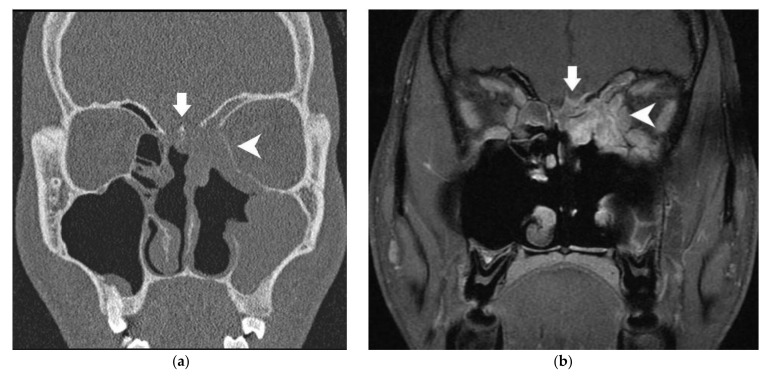
Twenty-year-old male subject with neuroendocrine carcinoma of the left ethmoid sinus. (**a**) Coronal CT scanner: minor erosion of the lamina papyracea (white arrowhead) and major erosion of the cribriform plate (white arrow). (**b**) Coronal MRI (T1 acquisition with fat saturation and gadolinium injection): white arrow: major modification of bony skull base, irregular nodular dural enhancement >2 mm, irregular deformation of the dura, contact angle ≤45°; white arrowhead: major modification of lamina papyracea, regular deformation of orbital content, invasion of the fat between tumor and oculomotor muscle. In the radiological conclusion, the bony skull base, orbital medial wall and dura were considered as invaded. Cerebral parenchyma and orbital content were considered as tumor-free.

**Figure 2:**
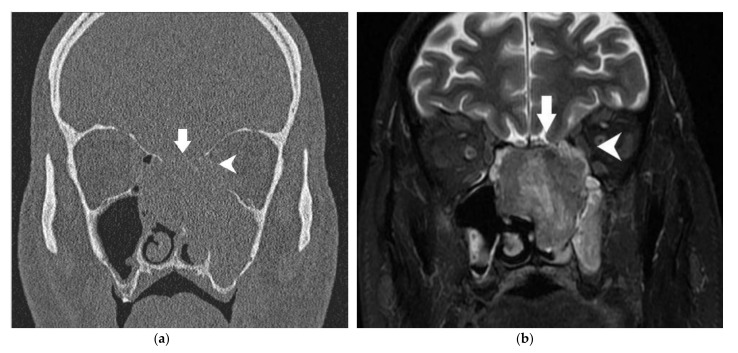
Sixty-three-year-old male subject with left ethmoido-maxillary non-intestinal-type adenocarcinoma. (**a**) Coronal CT scanner: major erosion of cribriform plate (white arrow) and lamina papyracea (white arrowhead). (**b**) Coronal MRI (T2 acquisition with fat saturation): white arrow: minor modification of bony skull base; white arrowhead: major modification of lamina papyracea, regular deformation of orbital content, invasion of the fat between tumor and oculomotor muscle. In the radiological conclusion, the bony skull base, dura, cerebral parenchyma, orbital bony walls and orbital content were all considered tumor-free.

**Figure A1:**
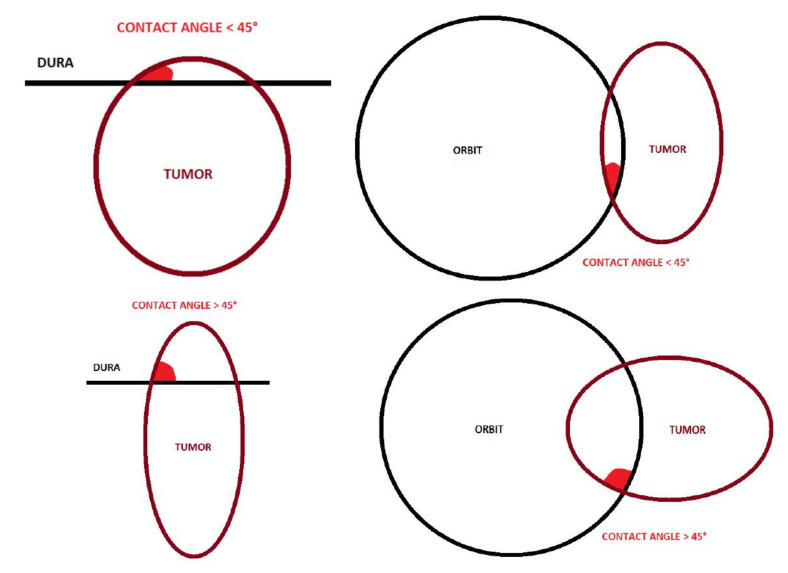
Assessment of contact angle of ≤45° or >45° between the tumor and dura or orbit.

Double prospective imaging reviewing was performed independently and blindly by two senior neuroradiologists of each center according to predefined radiological parameters designed for each anatomical structure, as described in Table 1. These parameters were determined based on the existing scientific literature and a collegial discussion between the neuroradiologists of both departments. On the CT scan, contact without modification of the bony skull base and/or the bony orbital wall was evaluated because this situation is, in our surgical algorithm, an indication to remove this structure. We wanted to evaluate the risk of invasion in these particular cases. In the literature, erosion of the skull base or the orbital wall on the CT scan is a common sign of invasion [10,11,12,13,14,15,20,21]. Likewise, on MRI, modification of the bony skull base/orbital bony wall (“black line”) [28] is associated with invasion [17]. However, their diagnostic performances are rarely assessed. We then distinguished minor (<2 mm) from major (≥2 mm) erosion on CT/modification on MRI given that minor erosion/modification is not always associated with pathologic invasion [29]. On MRI, dural enhancement is a common sign of dural invasion. Different patterns of enhancement (nodular, linear) [9,16] have been described. The thickness of the enhancement is also a crucial datum; previous studies suggest that the risk of invasion rises with the width (between 2 and 5 mm) [16,17,21]. Thus, we determined the cut-off value at ≤2 mm and >2 mm when linear and nodular enhancements were reported. On MRI, the correlation between edema of the brain parenchyma and tumor invasion is well described [13,20,29]. The aspect (smooth or irregular) of the deformation induced by the tumor on the dura or orbital content is reported in the literature, but its diagnostic performance has not been evaluated [28]. However, the contact angle of this deformation is not described in the literature, to our knowledge, but appeared relevant to be collected and assessed to our neuroradiologists. On MRI, the invasion of the intraorbital fat between the oculomotor muscles and tumor and the specific invasion of the oculomotor muscles have already been described [21,22]. Therefore, we chose to assess the diagnostic performance of these parameters. Figure 1 and Figure 2 present those radiological parameters in two radiological cases. In Appendix A (Figure A1), schematics are shown in order to define a “contact angle ≤45° or >45°” between the tumor and orbit or dura.

For the CT scan evaluation, only bone window CT slices were analyzed because iodinated contrast media injection was not routinely performed for sinonasal tumors in the two centers. T2 and contrast-enhanced T1 with fat saturation acquisitions in the axial and coronal planes were mandatory for the analysis of MRI procedures. If the quality of a radiological exam was not sufficient, the procedure was excluded.

For each patient and anatomical structure, radiologists had to conclude whether it was invaded or not by the tumor: this subjective evaluation was named “radiological conclusion”. In this cohort, olfactory bulb invasion was not assessed specifically on imaging given the heterogeneity of MRI acquisitions.

Imaging reviewing and radiological conclusion were compared to histopathological reports, which were considered the gold standard for the diagnosis of skull base/orbital invasion. In some cases, when the pathological analysis of certain anatomical structures was not available (for example, the periorbita), we considered it as non-invaded if it was described by the surgeon as macroscopically free of tumor.

Interobserver differences were gathered. For each difference, the final result was obtained by consensus after discussion between the radiologists.

Positive predictive value (PPV) and 95% confidence interval (IC95) were calculated for each parameter. Negative predictive value (NPV), sensitivity, specificity and accuracy were calculated for radiological conclusion. The strength of interobserver consensus for each parameter was determined by the kappa coefficient [30]. Yule’s Q was calculated for the radiological conclusion of each anatomical structure.

## 3. Results

### 3.1. Patients

Out of the 479 patients previously screened for “surgical treatment for sinonasal malignancy”, 303 were excluded because of incomplete available data (imaging, clinical data or histopathological reports). Thus, 176 patients were finally included in our study. The tumors originated mainly from the ethmoid sinus (88%). Most patients (74%) were diagnosed at advanced stages (T3/T4a–b). Intestinal-type sinonasal adenocarcinoma (ITAC) (41%) and esthesioneuroblastoma (20%) were the most common histological types. Seven patients (4%) required an orbital clearance. Patients’ characteristics are reported in Table 2. CT imaging was available for 140 patients (80%), and MRI was available for 160 patients (91%). CT combined with MRI was available for 125 patients. The mean delay between the CT scan and surgery was 51 days. The mean delay between MRI and surgery was 43 days.

### 3.2. Histopathological Data

Pathologic involvement of the bony skull base was reported in 78 patients (44%), with an associated dural invasion in 46 patients (26%). Among the 46 patients with dural invasion, pathologic involvement of the olfactory bulbs was reported in 54% (25/46). Esthesioneuroblastoma was the most common histological type (*n* = 10) in patients with olfactory bulb invasion, representing 28% of esthesioneuroblastoma cases overall. By comparison, five patients with intestinal-type adenocarcinoma had olfactory bulb invasion, representing 7% of all intestinal-type adenocarcinoma cases. Patients with esthesioneuroblastoma had a higher risk of olfactory bulb invasion compared with other histopathological types (*p* < 0.05). Orbital bony walls and orbital content were histologically invaded in 36 patients (20%) and 14 patients (8%), respectively.

### 3.3. Radiohistological Correlation

The diagnostic performances of CT and MRI for the diagnosis of skull base/orbital invasion are summarized in Table 3 and Table 4.

To assess the involvement of the bony skull base, major bone erosion on CT imaging had a PPV of 77.8%, while major modification on MRI had a PPV of 88.9%. Thus, the radiological conclusion for bony skull base invasion had a PPV of 76.9%, with a specificity of 83.7%.

Dural involvement was assessed on MRI. Nodular enhancement with a thickness superior to 2 mm, irregular deformation and a contact angle up to 45° obtained the highest PPVs, with 87.0%, 86.7% and 85.7%, respectively. However, in nine cases, the dura was considered as normal on MRI, whereas it was histologically invaded. In eight of these nine cases, the bony skull base was also normal on imaging. The commonest histological type among them was non-intestinal-type adenocarcinoma (*n* = 3), and other histological types were: esthesioneuroblastoma (*n* = 2), intestinal-type adenocarcinoma (*n* = 2), squamous cell carcinoma (*n* = 2). The mean delay between MRI and surgery was 46.7 days in this group of patients.

Only one patient with an ethmoidal esthesioneuroblastoma displayed a localized invasion of the cerebral parenchyma on MRI that was confirmed by histopathological examination. However, in five cases (3%), staged as T4b for a dural involvement, a cerebral invasion was not foreseen on MRI.

For orbital invasion, orbital bony walls and orbital content were assessed separately. Interestingly, CT assessment of the lamina papyracea and orbital floor had a better PPV (48.8%) than MRI (47.5%) for the diagnosis of orbital invasion. The radiological conclusion for orbital bony wall invasion obtained 80.6% sensitivity and 80.7% specificity. As for the intraorbital extension, no radiological sign seemed to have a significant predictive value. In the case of a contact angle up to 45° between the tumor and the periorbita, the periorbita was invaded in 45.4% of the cases. PPVs of the other parameters were inferior or equal to 33.3%. The radiological conclusion for orbital content invasion obtained a low sensitivity (28.6%) and PPV (50.0%).

The diagnostic accuracy of the radiological conclusion for each anatomical barrier is shown in Table 4. The lowest accuracy was 76.5% for orbital bony walls. Altogether, the accuracy of the radiological conclusion is usually over 80%, which is considered as satisfactory [31].

Kappa coefficients were mostly superior to 0.70, showing that the interobserver consensus was substantial for these data. Only three parameters obtained lower values with only a moderate interobserver consensus: cerebral parenchyma evaluation on MRI (κ = 0.52), radiological conclusion for cerebral parenchyma invasion (κ = 0.49) and orbital content invasion (κ = 0.41).

Yule’s Q for the radiological conclusion showed a strong (0.50–0.70) or a very strong (0.70–1) correlation with histopathological findings.

### 3.4. Comparison of cTNM and pTNM

Based on the pathological findings, the pT stage was modified in 64 cases (36%). Indeed, local extension had been overrated in 50 cases (28%), with ITAC representing half of the cases. Misinterpretation of bony skull base invasion on imaging was the commonest error.

Conversely, for 14 patients (8%), the local extension had been underrated. Misinterpretation of dural involvement on imaging was the commonest error.

Eleven patients (6%) were classified pT0 on histopathological reports. Among them, six had received neoadjuvant chemotherapy and five had been operated for diagnostic purposes prior to the oncological surgery.

Finally, cTNM was T3 or worse for 73% of the patients, whereas pTNM was T3 or worse for 54% of the patients.

## 4. Discussion

In this study, pre-therapeutic imaging efficiently assessed skull base and orbital invasion in sinonasal cancers. Nevertheless, some situations require pre-operative macroscopic evaluation and frozen section analysis, especially when orbital invasion is suspected. To our knowledge, this study depicts the largest series with a correlation between imaging features and histopathological findings in sinonasal cancer.

Patient and tumor characteristics were in accordance with the scientific literature: mean age of 57 years, male dominance, majority of advanced stage tumors and high incidence of ITAC [1]. All patients underwent partial or total anterior skull base resection, which is rare for this type of study. Only seven patients required orbital exenteration. This is consistent with the decrease in orbital clearance indications in the therapeutic algorithm, as previously reported [32]. Thus, histopathological analysis available for orbital content invasion was generally based on the periorbita.

In the case of dural invasion, olfactory bulbs were invaded for more than half of the patients. As expected, among patients with a tumor extension into the olfactory bulbs, esthesioneuroblastoma was the most common histological subtype. The olfactory bulbs can be difficult to distinguish on MRI if appropriate sequences are not available [33] or when the tumor invades the skull base on imaging. This outlines the relevance of resecting the olfactory bulbs when dural invasion is suspected, particularly in the case of esthesioneuroblastoma [4,34].

Cribriform plate, ethmoidal roof and planum invasion evaluated by imaging in this study obtained 76.9% sensitivity and 83.7% specificity, and these results are similar to those in the existing literature [20,21]. It was decided to distinguish minor from major erosion/modification. As expected, major erosion on CT imaging and major modification on MRI obtained high PPVs, whereas minor erosion or modification had lower PPVs. These thin bony structures can be stretched by tumor growth without being invaded at once; thus, minor or punctiform erosion may be visualized on imaging. Additionally, according to Singh et al. [29], it is common to see some lucent areas in the cribriform plate on CT imaging that can lead to an erroneous perception of cortical erosion.

On MRI, nodular enhancement with a thickness greater than 2 mm, irregular deformation and a contact angle greater than 45° were highly predictive of dural involvement (PPVs > 85%). To our knowledge, evaluation of the contact angle between the tumor and dura has not been reported in the literature until now. Dural invasion was not foreseen on imaging for nine patients in this study. Ziai et al. evaluated the occult rate of dural invasion among 37 patients presenting sinonasal malignancies with skull base encroachment without dural invasion on imaging: dural invasion was observed in seven patients [35]. Thus, extreme care must be taken at the time of pre-operative and intraoperative dural evaluation. Even though radiological dural evaluation shows a high NPV, occult dural invasion is not rare.

In this study, only few patients presented brain invasion. Hence, the validity of our results for this anatomical structure is limited. Although it is well accepted that the presence of an enhanced tumor in the brain parenchyma indicates brain invasion [13], brain edema alone does not necessarily imply parenchymal invasion [29]. This is confirmed in this study, as only half of the patients with edema of the cerebral parenchyma on MRI presented a histological brain invasion.

Pretreatment orbital assessment by imaging was ambivalent in this study. On one side, PPVs were inferior to 50% for each radiological sign, leading to the conclusion that imaging overestimated orbital invasion. On the other side, the radiological conclusion for orbital content invasion obtained an excellent NPV, specificity and accuracy, and only 4% of the patients required an orbital clearance because of a massive orbital intraconic invasion that was detected on pre-operative imaging. The literature is scarce on this subject [8,21,22,23,24]. Some authors [15,21,23] reported frequent contradictions between CT imaging, intraoperative and histological orbital evaluation. Tiwari et al. found five orbits without histopathological tumor invasion among 12 patients that underwent orbital exenteration, whereas pre-operative CT scans revealed orbital invasion [8]. MRI is considered as a better tool for orbital assessment. According to Kim et al. [24], in a series of 10 patients, invasion of the orbital fat on MRI is predictive of orbital invasion. Eisen et al. [22] studied MRI performance to assess orbital invasion in 25 patients with sinonasal tumors: extra-conic fat involvement and extra-oculomotor muscle modification obtained, respectively, 80% and 100% PPVs, but no radiological criteria were statistically associated with orbital invasion. Meerwein et al. [21] evaluated medial orbital wall infiltration on imaging in a large series of sinonasal cancers: they also found a high rate of false positive cases, emphasizing the need for intraoperative exploration to accurately determine medial orbital wall infiltration. The properties of orbital anatomical structures explain these results; the lamina papyracea is a thin bony structure easily invaded, but the periorbita is thick and deformable, being able to contain the tumor extension. Moreover, some slow-growing tumors tend to push back adjacent anatomical structures without invading it at once [21]. Thus, during surgery, we frequently observe tumors arising from the olfactory cleft that compress the middle turbinate and ethmoid cells laterally against the lamina papyracea. In this situation, imaging generally shows orbital abnormalities with an absence of the medial orbital wall, while intraoperative and histopathological findings are reassuring [36]. Thus, the absence of the lamina papyracea could be due to the local hyperpressure induced by the tumor or the peritumoral inflammation. These results support the actual trend limiting the indications of orbital clearance [32,37].

T classification was modified after surgery for 36% of the patients, which is comparable with other studies [21,38]. Local extension of the tumor had been overrated in most cases. This is consistent with our results: PPVs were sometimes moderate, especially for orbital evaluation. Consequently, a small proportion of patients underwent unnecessary skull base and/or dural resection. On the other hand, local tumor extensions were underrated in some cases, especially dural involvement. As NPVs did not reach 100%, especially for skull base invasion (82.0%), it is not possible to rule out skull base or dural invasion when imaging is considered as “normal”. This outlines the limit of imaging in sinonasal tumors: radiological sensibility was 78.6% for assessing dural invasion in this study. In some cases, the delay between imaging and surgery may be considered as long, which can constitute a study limitation. Thus, to limit the bias of a potential evolution between the MRI and surgical procedures, or in case of a fast-growing tumor, it could be useful to perform MRI the day before surgery. Finally, even with a complete imaging assessment, it is essential, as a first surgical step, to evaluate the tumoral extensions with trans-nasal endoscopy, eventually after debulking, to confirm the adequate surgical procedure and reconstruction technique.

The retrospective nature of this study is a limitation, as a certain subset of patients had to be excluded because of a lack of data, especially imaging data. Due to the study design, we only included patients who underwent surgery, leading to a potential selection bias. Furthermore, despite a large number of patients, only a few patients presented orbital and cerebral invasion, limiting the statistical power of our conclusions for these anatomical structures. The histological heterogeneity observed in this study is characteristic for sinonasal tumors. Even though analysis of each histological type separately seems logical, the low incidence of some histological subtypes makes it difficult. Only bony window CT scans were studied in this study because they are considered as a complement to MRI to assist surgery. The performance of the CT scan could, therefore, not be extensively evaluated. On MRI, radiological signs were not specific to certain MRI sequences. Yet, according to Kim et al. [24], periorbita invasion is better assessed on T2 sequences because the signal of the tumor is better visualized.

## 5. Conclusions

This retrospective study provides objective data about the diagnostic value of pretreatment imaging in patients with resectable sinonasal cancer. In particular, it suggests that pretreatment assessment of orbital invasion is difficult, even with the combination of CT and MRI.

## Figures and Tables

**Table 1:** Radiological parameters.

Anatomical Structure	Imaging	Radiological Sign	Radiological Conclusion
Bony skull base	CT scanner	Contact without bony modification Minor (<2 mm) erosion Major (≥2 mm) erosion	Free Invaded
	MRI	Minor (<2 mm) modification Major (≥2 mm) modification	
Dura	MRI	Linear enhancement: ≤2 mm or >2 mm Nodular enhancement: ≤2 mm or >2 mm Smooth or irregular deformation Contact angle: ≤45° or >45°	Free Invaded
Cerebral parenchyma	MRI	Edema Tumoral invasion	Free Invaded
Orbital bony walls	CT scanner	Contact without bony modification Minor (<2 mm) erosion Major (≥2 mm) erosion	Free Invaded
	MRI	Minor (<2 mm) modification Major (≥2 mm) modification	
Orbital content	MRI	Invasion of the fat between tumor and oculomotor muscle Smooth or Irregular deformation Contact angle: ≤45° or >45° Invasion of oculomotor muscle	Free Invaded

**Table 2:** Patient and tumor characteristics.

Patients (Number)	176
Mean age in years (extremes)	57 (16–89)
Sex ratio (Male/Female)	3.8 (139/37)
Side of the tumor (number and percentage) Bilateral Right Left	2 (1%) 89 (51%) 85 (48%)
Primitive location (number and percentage) Ethmoid Olfactory cleft Nasal septum Middle turbinate Frontal Maxillary bone Orbit Sphenoid	155 (88%) 13 (7%) 3 (2%) 1 1 1 1 1
Histological type (number and percentage) Intestinal-type adenocarcinoma Esthesioneuroblastoma Non-intestinal-type adenocarcinoma Squamous cell carcinoma Mucosal melanoma Neuroendocrine carcinoma Rhabdomyosarcoma Other	72 (41%) 36 (20%) 28 (16%) 10 (6%) 9 (5%) 6 (3%) 3 (2%) 12
Pre-operative T classification Tx T1 T2 T3 T4a T4b	8 (5%) 3 (2%) 36 (20%) 47 (27%) 23 (14%) 58 (33%)
Pre-operative N classification N0 N1 N2b N2c Retropharyngeal	168 (95%) 13 (2%) 3 (2%) 1
Post-operative T classification T0 T1 T2 T3 T4a T4b	11 (6%) 5 (3%) 65 (37%) 23 (13%) 16 (9%) 56 (32%)
First tumor Recurrence	169 (96%) 7 (4%)
Pre-operative treatments (number and percentage) Chemotherapy Radiochemotherapy	66 (38%) 1
Surgical technique (number and percentage) Craniofacial resection Endoscopic trans-nasal resection Cranio-endoscopic resection	58 (33%) 117 (66%) 1
Post-operative treatments (number and percentage) Exclusive radiotherapy Radiochemotherapy	149 (85%) 10 (6%)
Histological invasion: Bony skull base Dura Olfactory bulbs Cerebral parenchyma Bony orbital walls Orbital content	78 (44%) 46 (26%) 25 (14%) 9 (5%) 36 (20%) 15 (9%)

**Table 3:** Radiohistological correlation.

Anatomical Structure	Imaging	Radiological Sign		Number of Abnormalities	PPV (CI95)	Kappa
Bony skull base	CT scanner	Contact without bony modification		41	7.3% (1.5–19.9)	0.87
		Minor erosion		32	50.0% (32.7–67.3)	
		Major erosion		54	77.8% (66.7–88.9)	
	MRI	Minor modification		40	45.0% (29.6–60.4)	0.85
		Major modification		45	88.9% (79.7–98.1)	
Dura	MRI	Linear enhancement	≤2 mm	39	38.5% (23.2–53.7)	0.78
			>2 mm	14	71.4% (41,9–91.6)	
		Nodular enhancement	≤2 mm	5	40.0% (5.3–85.3)	0.79
			>2 mm	23	87.0% (66.4–97.2)	
		Deformation	Smooth	41	46.3% (31.1–61.6)	0.73
			Irregular	15	86.7% (59.5–98.3)	
		Contact angle	≤45°	35	37.1% (21.1–53.2)	0.84
			>45°	21	85.7% (63.7–97.0)	
Cerebral parenchyma	MRI	Edema		4	50.0% (6.8–93.2)	0.52
		Tumoral invasion		1	100% (2.5–100)	
Orbital bony walls	CT scanner	Contact without bony modification		47	0% (0–7.6)	0.85
		Minor erosion		22	27.3% (10.7–50.2)	
		Major erosion		43	48.8% (33.9–63.8)	
	MRI	Minor modification		21	28.6% (11.3–52.2)	0.90
		Major modification		40	47.5% (32.0–63.0)	
Orbital content	MRI	Invasion of the fat between tumor and oculomotor muscle		24	33.3%(15.6–55.3)	0.91
		Deformation	Smooth	45	28.6% (13.6–43.5)	0.75
			Irregular	6	33.3% (4.3–77.7)	
		Contact angle	≤45°	40	21.2% (7.3–35.2)	0.80
			>45°	11	45.5% (16.8–76.6)	
		Invasion of oculomotor muscle		3	33.3% (0.8–90.6)	1

Minor erosion/modification: <2 mm; major erosion/modification: ≥2 mm.

**Table 4:** Radiological conclusion.

Radiological Conclusion	Bony Skull Base	Dura	Cerebral Parenchyma	Orbital Bony Walls	Orbital Content
True positives	60	33	1	29	4
True negatives	82	97	151	113	142
False positives	16	21	1	27	4
False negatives	18	9	7	7	10
Sensitivity (CI95)	76.9% (67.6–86.3)	78.6% (66.2–91.0)	12.5% (0.3–52.7)	80.6% (67.6–93.5)	28.6% (8.4–58.1)
Specificity (CI95)	83.7% (76.4–91.0)	82.2% (75.3–89.1)	99.3% (98.1–100)	80.7% (74.2–87.3)	97.3% (94.6–99.9)
PPV (CI95)	79.0% (69.8–88.1)	61.1% (48.1–74.1)	50.0% (1.3–98.7)	51.8% (38.7–64.9)	50.0% (15.4–84.7)
NPV (CI95)	82.0% (74.5–89.5)	91.5% (86.2–96.8)	95.6% (92.4–98.8)	94,2% (90.0–98.4)	93.4% (89.5–97.4)
Accuracy (CI95)	80.7% (74.9–86.5)	81.3% (75.2–87,3)	95.0 (91.6–98.4)	76.3% (70.2–82.5)	91.3% (86.9–95.6)
Kappa	0.87	0.96	0.49	0.93	0.41
Yule’s Q	0.61	0.89	0.91	0.61	0.87

For the dura, cerebral parenchyma and orbital content, only 160 patients were included because MRI was only available for 160 patients.

## Data Availability

Data available on request due to ethical restrictions.

## Appendix A

These schematics are shown in order to illustrate the contact angle of ≤45° or >45° between the tumor and dura or orbit.
